# The altered gut virome community in rhesus monkeys is correlated with the gut bacterial microbiome and associated metabolites

**DOI:** 10.1186/s12985-019-1211-z

**Published:** 2019-08-19

**Authors:** Heng Li, Hongzhe Li, Jingjing Wang, Lei Guo, Haitao Fan, Huiwen Zheng, Zening Yang, Xing Huang, Manman Chu, Fengmei Yang, Zhanlong He, Nan Li, Jinxi Yang, Qiongwen Wu, Haijing Shi, Longding Liu

**Affiliations:** 1 Institute of Medical Biology, Chinese Academy of Medical Sciences & Peking Union Medical College, Kunming, 650118 China; 2 Key Laboratory of Systemic Innovative Research on Virus Vaccine, Chinese Academy of Medical Sciences, Kunming, China

**Keywords:** Gut viral community, Rhesus monkeys, Metagenomic analysis, Bacterial microbiome, Metabolite analysis, Correlation

## Abstract

**Background:**

The gut microbiome is closely associated with the health of the host; although the interaction between the bacterial microbiome and the whole virome has rarely been studied, it is likely of medical importance. Examination of the interactions between the gut bacterial microbiome and virome of rhesus monkey would significantly contribute to revealing the gut microbiome composition.

**Methods:**

Here, we conducted a metagenomic analysis of the gut microbiome of rhesus monkeys in a longitudinal cohort treated with an antibiotic cocktail, and we documented the interactions between the bacterial microbiome and virome. The depletion of viral populations was confirmed at the species level by real-time PCR. We also detected changes in the gut metabolome by GC-MS and LC-MS.

**Results:**

A majority of bacteria were depleted after treatment with antibiotics, and the Shannon diversity index decreased from 2.95 to 0.22. Furthermore, the abundance-based coverage estimator (ACE) decreased from 104.47 to 33.84, and the abundance of eukaryotic viruses also changed substantially. In the annotation, 6 families of DNA viruses and 1 bacteriophage family were present in the normal monkeys but absent after gut bacterial microbiome depletion. Intriguingly, we discovered that changes in the gut bacterial microbiome composition may promote changes in the gut virome composition, and tryptophan, arginine, and quinone may play roles in the interaction between the bacterial microbiome and virome.

**Conclusion:**

Our results indicated that the clearly altered composition of the virome was correlated with depletion in the bacterial community and that metabolites produced by bacteria possibly play important roles in the interaction.

**Electronic supplementary material:**

The online version of this article (10.1186/s12985-019-1211-z) contains supplementary material, which is available to authorized users.

## Background

The gut microbiome of an animal consists of bacteria, viruses, fungi and so on. This intricate ecosystem interacts with the adjacent epithelial layer, and the microbes perform metabolic functions, protect against pathogens, and condition the immune system, and through these basic functions, these microbes directly or indirectly affect most of the physiological functions of the host [1, 2]. In recent years, variations in the bacterial community composition have been shown to correlate with infection outcome, inflammatory bowel disease [3], diabetes [4], obesity [5], and depression [6], and fecal microbiome transplantation has become an effective treatment for refractory *Clostridium difficile* infections and other diseases [7, 8]. The mechanisms of interaction between the gut bacterial microbiome and the host are very complex, and other components also play crucial roles in this process. In addition to bacteria, viruses are also abundant in the gut [9] and have been hypothesized to markedly alter the structure and function of the bacterial community [10–12]. Additionally, chronic viral infection can confer increased resistance against pathogenic challenges [13]. Gut virome alteration has been observed in inflammatory diseases such as inflammatory bowel disease and Crohn’s disease [14]. The recent advent of high-throughput sequencing methods has made it possible to study these communities and their relationships with health and disease in detail [15].

Bacterial communities play an essential role in host health, but further research is still warranted to obtain an in-depth understanding of the mechanisms underlying this role. Transfer of whole virome communities between humans was documented in fecal microbiome transplantation [16], and the difference varied more widely between gut viromes than between gut bacterial microbiomes in humans [17]. However, the relationship between the bacterial microbiome and the virome has rarely been studied, despite its likely medical importance. Previous research has shown close relationships between single viral species and single bacterial species [18, 19], and single viral species could trigger shifts in the bacterial microbiome and the virome [20, 21]. At the same time, enteric bacteria were seen to be required for efficient infection by [22, 23] or suppression of [24] viruses, and the richness of the gut bacterial microbiome had an obvious effect on bacteriophage composition [25]; moreover, the gut virome composition in humans was examined, and bacteriophage diversity was found to be inversely correlated with naturally occurring bacterial diversity in human infants during healthy development [26]. However, few studies have focused on how the whole virome, a diverse community consisting of eukaryotic RNA and DNA viruses and bacteriophages, interacts with the bacterial microbiome.

Rhesus monkeys are good mammalian research models that are closely related to humans, and the virome composition of these animals was seen to be affected by simian immunodeficiency virus infection [20]. We hypothesized that there is a close relationship between the whole gut virome and bacterial microbiome, and the bacterial microbiome could be depleted by treatment with an antibiotic cocktail in rhesus monkeys. We then examined the virome composition to detect the direct effects of the bacterial microbiota on the virome. We performed 16S rRNA amplicon sequencing of the fecal bacteria and metagenomic analysis of fecal viromes from rhesus monkeys treated with an antibiotic cocktail. Our results suggest that a majority of bacteria were depleted after the monkeys were treated with antibiotics and that the composition of the whole virome changed drastically. Importantly, alteration of the virome along with shifts in the composition and function of the gut bacterial community and metabolites from gut bacteria may have played an important role in the interaction.

## Materials and methods

### Animals

The rhesus monkey cohort described in this study was housed at the Institute of Medical Biology, Chinese Academy of Medical Sciences (IMBCAMS). An antibiotic cocktail containing ampicillin, streptomycin, kanamycin, metronidazole, and vancomycin was administered orally at a dose of 15 mg/kg 3 times per day for 2 weeks. Three healthy one-year-old rhesus monkeys were treated with antibiotics, and fresh fecal samples were collected one day before treatment with antibiotics and 5, 8, and 9 days after treatment with antibiotics and stored at − 80 °C for subsequent analysis. Fresh fecal samples from an additional three normal monkeys were collected after the monkeys were treated with antibiotics for 9 days. The bacterial community of each sample was detected by 16S rRNA amplicon sequencing, and the virome communities in the samples collected before treatment with antibiotics and in those collected after treatment with antibiotics for 9 days were detected by deep sequencing. Because metabolome analysis requires 6 biological duplications, the metabolomes of samples collected before treatment with antibiotics and of those collected after treatment with antibiotics for 8 and 9 days were detected by GC-MS and LC-MS. In our analysis, samples collected before treatment with antibiotics were used as the control group, and samples collected after treatment with antibiotics were used as the experimental group.

### Bacterial 16S rRNA amplicon sequencing

DNA was extracted from fecal samples, and PCR was performed with the barcode primers 338F/806R to obtain amplicons of hypervariable regions V3 and V4 for phylogenetic discrimination analysis [27]. Libraries were pooled by using a TruSeqTM DNA Sample Prep Kit and sequenced using an Illumina MiSeq sequencer. Sequences were assigned to closed-reference operational taxonomic units (OTUs) at a 97% identity threshold using bacterial 16S rRNA amplicon sequences from the Silva 128/16S-bacteria database. The OTU data were rarefied to the smallest effective sample sizes [28]; rarefaction is a homogenization method that is used to randomly draw OTUs to the same quantity based on a minimum value. The α diversity, which includes the abundance-based coverage estimator (ACE) and Shannon diversity index, was analyzed by Mothur (version v.1.30.1), and statistical significance was evaluated by Student’s t-test.

### Kyoto encyclopedia of genes and genomes (KEGG) prediction analysis of the bacterial microbiome [29, 30]

We performed a Kyoto Encyclopedia of Genes and Genomes (KEGG) prediction analysis of the bacterial microbiome using PICRUSt. PICRUSt contains the Cluster of Orthologous Groups of proteins (COG) and KEGG Ortholog (KO) information corresponding to Greengene ID numbers. For metagenome prediction, PICRUSt takes an input OTU table containing identifiers that match tips from the marker gene with corresponding abundances for each of the OTUs across one or more samples. First, PICRUSt normalizes the OTU table based on 16S rRNA amplicon copy number prediction so that the OTU abundances accurately reflect the true abundances of the underlying organisms. The metagenome is then predicted by looking up the precalculated genome content for each OTU, multiplying the normalized OTU abundance by each KO abundance in the genome and summing these KO abundances together per sample. The prediction yields a table of KO abundances for each metagenome sample in the OTU table.

### Analysis of similarities (ANOSIM)

Analysis of similarities (ANOSIM) is a nonparametric test that shows whether the difference between groups is greater than that within groups. The analyses were performed in vegan or QIIME in R (version3.2.2) by using the Bray-Curtis algorithm [31].

### Virome DNA and RNA purification and sequencing

Fecal samples were suspended in phosphate-buffered saline (PBS) and filtered through a filter with a pore size of 0.45 μm (Millipore). The supernatant was enriched by a 30-kDa molecular mass filter (Ultra-15 30 K, Millipore). The concentrate was treated with DNase I (TaKaRa) at 37 °C for 30 min to eliminate unencapsulated nucleic acids. Subsequently, total viral DNA was extracted from half of the concentrate using the QIAamp DNA Stool Kit (Qiagen), and at the same time, total viral RNA was extracted from the other half using the QIAamp Viral RNA Kit (Qiagen). The extracted RNA was synthesized into double strands using the NEBNext RNA First Strand Synthesis Module (NEB) and the NEBNext mRNA Second Strand Synthesis Module (NEB). The DNA and double-stranded cDNA were amplified by whole-genome amplification (REPLI-g Mini Kit, Qiagen) and then fragmented into approximately 300-bp fragments by a Covaris M220 instrument. Then, the fragments were amplified into a PE library by the TruSeq™ DNA Sample Prep Kit and fixed to the chip by bridge PCR using the HiSeq 3000/4000 PE Cluster Kit. The constructs were sequenced on the Illumina HiSeq platform using HiSeq 3000/4000 SBS Kits.

For virome analysis, we first sheared the adaptor sequences with Seqprep and removed reads that were shorter than 50 bp and those that contained N bases with Sickle to retain the paired-end reads and single-end reads. We compared these reads to the host (rhesus monkey) genome by BWA and removed the reads belonging to the host. Then, we compared all the clean reads with the U.S. National Center for Biotechnology Information (NCBI) Nucleotide database to identify the sequences that belonged to viruses and the sequences that did not belong to any known genome, such as those of bacteria, fungi or other known microorganisms. Then, contigs were built from these reads. The contigs and all reads that could not be mapped to any known genome in NCBI were compared with the virus protein database in the NCBI nonredundant RefSeq database (including sequences from SwissProt, PIR, PRF, and PDB and coding sequences (CDS) from GenBank and RefSeq) based on amino acid sequences using BLASTP (BLAST version 2.2.31+, e-value: 1e-5). These results constituted our virus database and were used to obtain the nonredundant gene catalog by CD-HIT. All the reads were compared to our virus database to analyze their richness. The spliced read alignments were predicted by MetaGene, compared to the EggNOG database and Virulence Factors database (VFDB) for COG analysis using BLASTP (BLAST version 2.2.31+, e-value: 1e-5) and annotated using VFDB.

### PCR validation

The abundance results that were similar in 2 or more monkeys were selected, and real-time PCR was used to validate the changes in these viruses (SYBR Premix Ex Taq II, TaKaRa). As the template, we used the DNA and cDNA extracted from fecal samples. The samples that were not detected directly from the DNA or cDNA were subjected to multiple displacement amplification (MDA) (total nucleic acid was amplified by multiple displacement to comprehensively detect both DNA and RNA viruses [26]) and then analyzed by real-time PCR. For real-time PCR, we used the Ct numbers to show the richness of the virus. Viruses that were not detected were not shown. Primers were designed to amplify specific regions in the *Bdellovibrio* phage phiMH2K (5′-AATCCTCAATTCCAGACTTCCA-3′ (F) and 5′-CCATTTCCATAAGTCCGAGTG-3′ (R)), Bacillus phage B103 (5′- TGGCGATGTTGATGATGAC-3′ (F) and 5′-CTTTATTTGCGTCTGTTGTCG-3′ (R)), columbid circovirus (5′-TCAGGAGACGAAGGACACG-3′ (F) and 5′- TGGCATCATACATCGGGAC-3′ (R)), potato virus M (5′-CGCTTCGCTGCTTTCG − 3′ (F) and 5′-CGGACCATTCATACCACCA-3′ (R)), *Marseillevirus marseillevirus* (5′-AAAGTCCCAAGTTATCACAAGC-3′ (F) and 5′- TTTCTCGCAGCGTCAATG-3′ (R)), simian sapelovirus (5′- TTCCATCTGCTCTAAATGCTCA-3′ (F) and 5′-CAGCAGTTAGAGCGGGTG-3′ (R)), and Andean potato mild mosaic virus (5′-AAGCCCAACATCGTTCTCC-3′ (F) and 5′- AAGAGGATACGGGAGAAAGG-3′ (R)).

### Redundancy analysis (RDA)

Redundancy analysis (RDA) shows the interactions between sample distribution and environmental factors. We used vegan’s RDA analysis in R with the phylum-level abundances of the bacterial microbiome as environmental factors.

### Regression analysis

We ran a regression analysis between bacterial microbiome diversity and virome richness with the stats package and plotted the results using the ggplot2 package.

### Metabolome detection

The stool samples were suspended in methanol:H_2_O (4:1), ground, ultrasonicated, concentrated and dried so that the metabolome could be analyzed by GC-MS and LC-MS.

#### GC-MS

The derivatized samples were analyzed on an Agilent 7890A gas chromatography system coupled to an Agilent 5975C MSD system (Agilent). An HP-5 MS fused-silica capillary column (30 mm × 0.25 mm × 0.25 μm, Agilent) was utilized to separate the derivatives. Helium (> 99.999%) was used as the carrier gas at a constant flow rate of 6.0 mL/min through the column. The injector temperature was maintained at 280 °C. A volume of 1 μL was injected in splitless mode. The oven temperature was initially 60 °C and was then ramped up to 125 °C at a rate of 8 °C/min, to 190 °C at a rate of 10 °C/min, to 210 °C at a rate of 4 °C/min and to 310 °C at a rate of 20 °C/min; finally, the temperature was held at 310 °C for 8.5 min. The temperatures of the MS quadrupole and ion source (electron impact) were set to 150 °C and 230 °C, respectively. The collision energy was 70 eV. Mass data were acquired in full-scan mode (m/z 50–600), and the solvent delay time was set to 5 min. The acquired MS data from GC-MS were analyzed by ChromaTOF software (v 4.34, LECO, St Joseph, MI). Metabolites were qualitatively assessed by the Fiehn database, which is linked to ChromaTOF software. Briefly, after alignment with the Statistic Compare component, a CSV file was obtained with three-dimensional data sets, including sample information, peak name, retention time, m/z and peak intensities. The resulting data were normalized to the total peak area of each sample in Excel 2007 (Microsoft, USA) and imported into SIMCA (version 14.0, Umetrics, Umeå, Sweden) to define the 95% confidence interval of the modeled variation. The differential metabolites were selected on the basis of the combination of a statistically significant threshold of variable influence on projection (VIP) values obtained from the OPLS-DA model and *p* values from a two-tailed Student’s t-test on the normalized peak areas, where metabolites with VIP values larger than 1.0 and *p* values less than 0.05 were included.

#### LC-MS

LC-MS was performed on an Ultimate 3000-Velos Pro system equipped with a binary solvent delivery manager and a sample manager coupled with an LTQ Orbitrap mass spectrometer equipped with an electrospray interface (Thermo Fisher Scientific); an Acquity BEH C18 column (100 mm × 2.1 mm i.d., 1.7 μm; Waters) was used. The column was maintained at 45 °C, and separation was achieved using the following gradient: 5% B–25% B from 0 to 1.5 min, 25% B–100% B from 1.5 to 10.0 min, 100% B from 10.0 to 13.0 min; 100% B–5% B from 13.0 to 13.5 min, and 5% B from 13.5 to 14.5 min at a flow rate of 0.40 mL/min, where B was acetonitrile (0.1% (v/v) formic acid), and A was aqueous formic acid (0.1% (v/v) formic acid). The injection volume was 3.00 μL, and the column temperature was set at 45.0 °C. The mass spectrometric data were collected using an LTQ Orbitrap mass spectrometer equipped with an electrospray ionization (ESI) source operating in either positive or negative ion mode. The capillary and source temperatures were set at 350 °C, with a desolvation gas flow of 45 L/h. Centroid data were collected from 50 to 1000 m/z with a resolution of 30,000. XCMS (http://masspec.scripps.edu/ xcms/xcms.php) was used for nonlinear alignment of time domain data and automatic integration and extraction of the peak intensities. Default XCMS parameter settings were used (major default parameters: profmethod = bin; method = matched filter; step = 0.1) except for full width at half maximum = 8, bandwidth (bw) = 6 and snthresh = 5. Variables with < 30% relative standard deviation (RSD) in QC samples were then retained for further multivariate data analysis. The result was a three-dimensional matrix that included retention time and m/z pairs (variable indices), sample names (observations), and normalized ion intensities (variables). The positive and negative data were merged into a combined data set, which was imported into SIMCA-P+ 14.0 software (Umetrics, Umeå, Sweden). The differential metabolites were selected on the basis of a combination of statistically significant VIP values obtained from the OPLS-DA model and *p* values from a two-tailed Student’s t-test on the normalized peak areas, where metabolites with VIP values larger than 1.0 and p values less than 0.05 were included. The differential metabolites were qualitatively assessed using the Human Metabolome Database (http://www.hmdb.ca/) and METLIN (https://metlin.scripps.edu/).

## Results

**Fig. 1:**
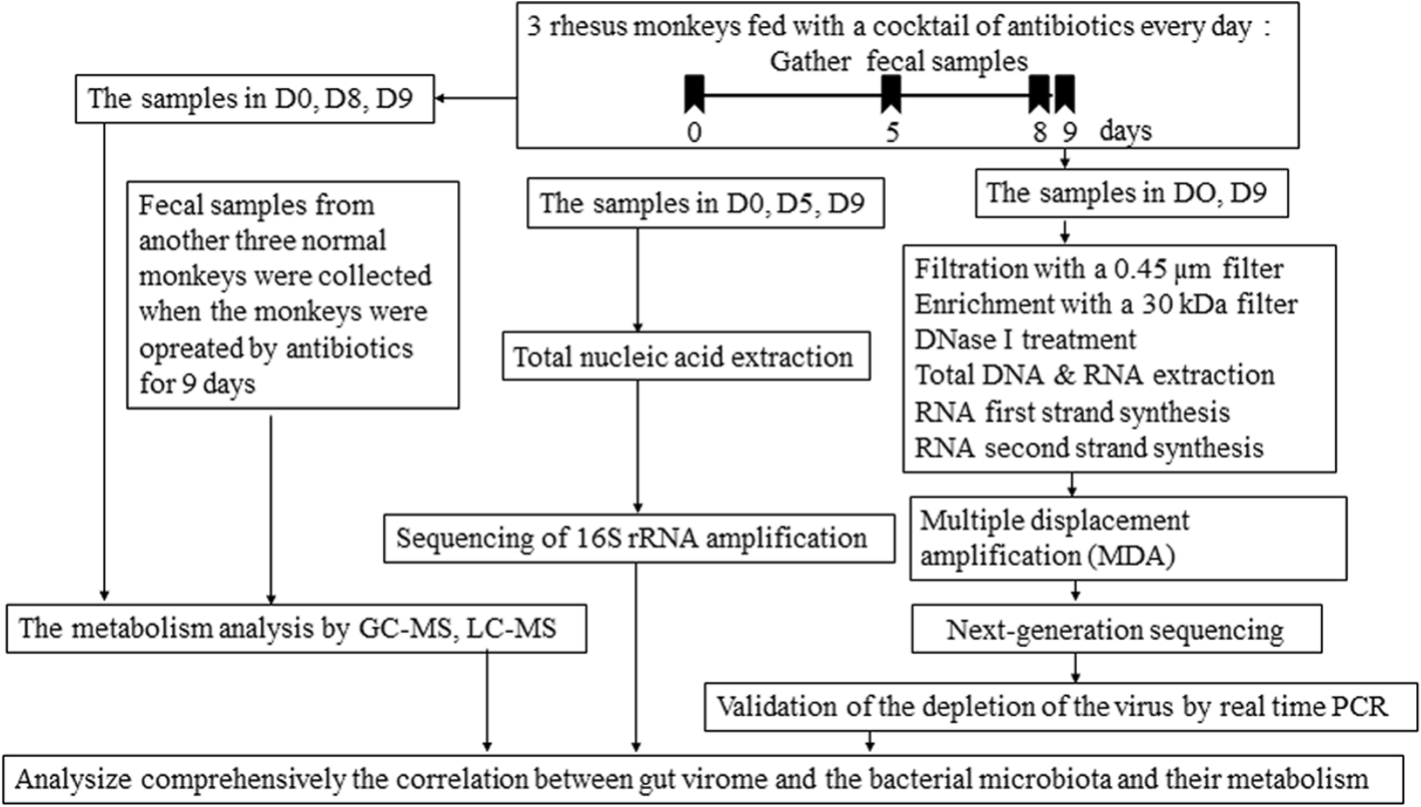
Experimental procedure. Three rhesus monkeys were treated with an antibiotic cocktail to control their gut bacterial microbiome, and we detected the longitudinal changes in the gut bacterial microbiome at D0, D5 and D9 by 16S rRNA amplicon sequencing. Then, we extracted nucleic acids from the fecal supernatant at D0 and D9 and scanned the gut viromes of the monkeys. The samples for metabolomics were collected on D0, D8 and D9 and scanned by GC-MS and LC-MS. We comprehensively analyzed the interactions among the gut virome, bacterial microbiome and metabolomes based on the above results

We performed metagenomic sequencing of fecal samples to detect the bacterial microbiome and virome composition of healthy one-year-old rhesus monkeys housed at the IMBCAMS (Fig. 1). The rhesus monkeys were monitored by blood cell analysis, which is the examination of blood condition and disease by observing the number and distribution of blood cells during the course of antibiotic treatment [32], and we found no obvious differences between the normal monkeys and those treated with antibiotics (Additional file 1: Figure S1).

### The bacterial microbiome was depleted stably and continuously by antibiotic treatment

The composition of the bacterial microbiome was investigated by extracting DNA directly from feces for 16S rRNA gene amplification. We used the hypervariable regions V3 and V4 to perform phylogenetic discrimination with the barcode primers 338F/806R [27]. In total, 556,012 amplicon reads (37,067 ± 6872 per sample) were obtained.

**Fig. 2:**
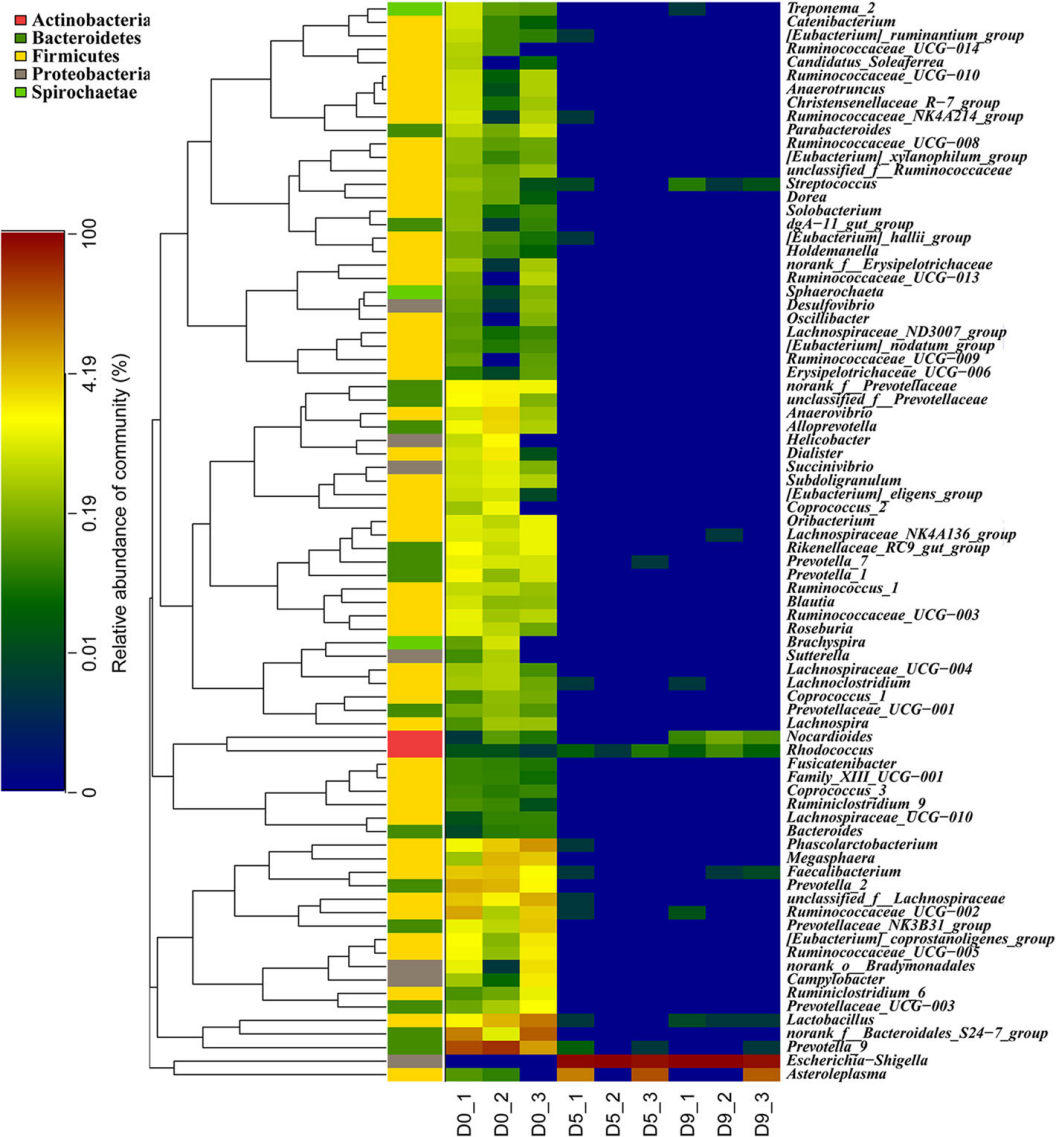
The bacterial microbiome was obviously depleted by treatment with antibiotics. Heatmap of the OUT percentage of every genus at 3 timepoints. Each point represents 3 biological replicates. The genera that belong to the same phylum are shown in the same color on the left. The total OTU number of 3 biological replicates for every genus was more than 10. The color bar represents the log of the percentage, the numbers in the heatmap are the log values of the OTU numbers, and the numbers in the bar are the percentages

**Fig. 3:**
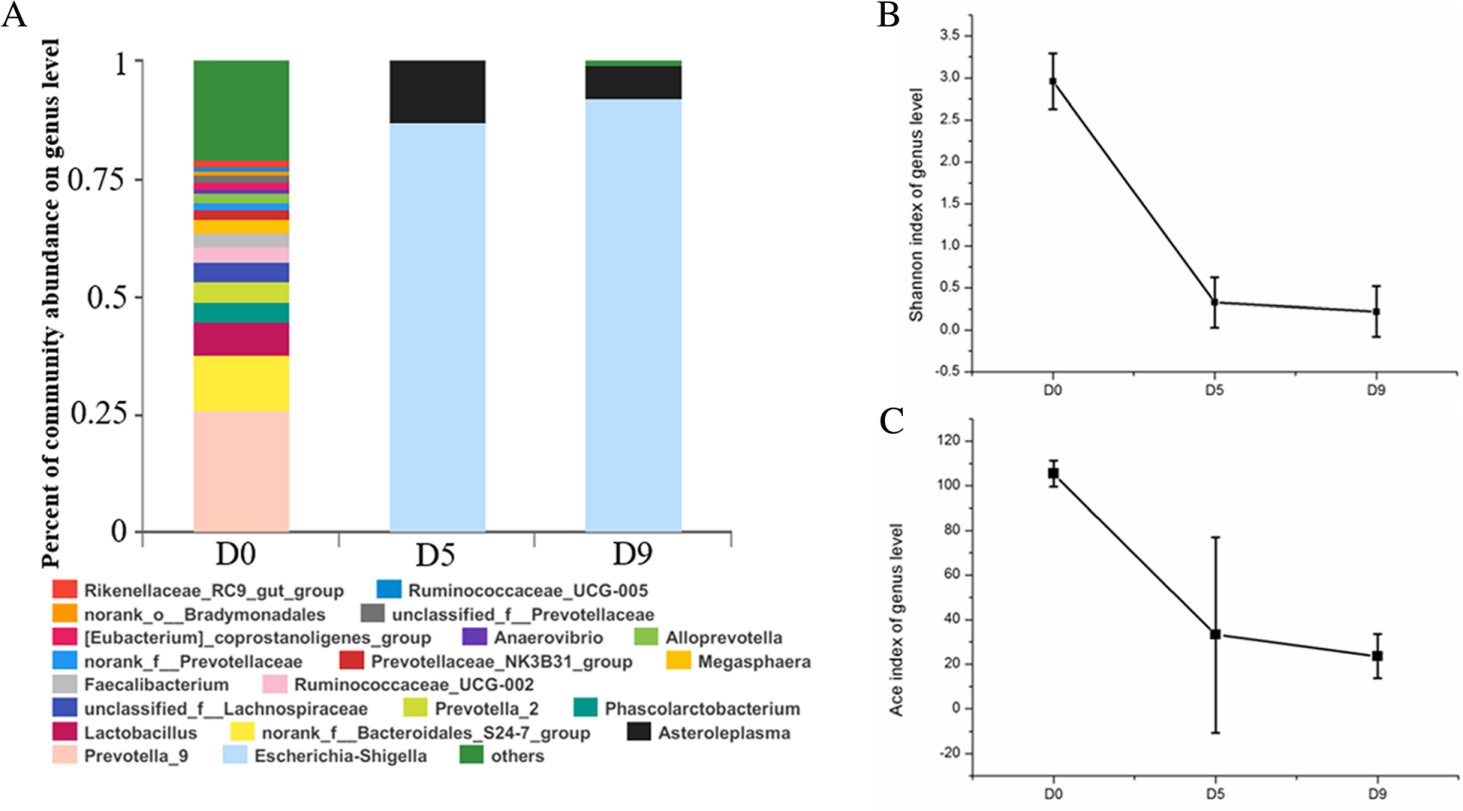
The bacterial microbiome was depleted stably and continuously by treatment with antibiotics. (**a**) Percentage of community abundance at the genus level at each timepoint. The bacterial composition changed noticeably, and the diversity of the bacterial microbiome decreased sharply. We took the average of the 3 biological replicates at each timepoint, and every genus is presented in its own color. (**b**, **c**) α-Diversity analysis of the bacterial microbiome calculated by Mothur (version v.1.30.1). The Shannon diversity index represents the diversity of the bacterial microbiome, and the ACE shows richness. We show the average of the 3 biological replicates at each timepoint

At the phylum level, the fecal bacterial communities were composed predominantly of high abundances of Bacteroidetes (53.1%) and Firmicutes (42%) and low levels of Proteobacteria (3.82%) (Additional file 2: Figure S2A). As expected, the enteric bacterial microbiome was depleted significantly after exposure to antibiotics (Fig. 2, 3a; Additional file 2: Figure S2, Additional file 3: Table S1, Additional file 4: Figure S3). The levels of Firmicutes and Bacteroidetes decreased to 7.69% and below 0.01%, respectively; at the same time, the abundance of Proteobacteria increased to 92% (Additional file 2: Figure S2B). *Escherichia*-*Shigella* were the major constituents of Proteobacteria, and other genera belonging to Proteobacteria were markedly depleted (Additional file 2 : Figure S2B, Additional file 3: Table S1). The fecal samples were spread on plates with the antibiotic cocktail, and a large number of colony-forming units (CFUs) were observed in the sample from D9 but not in the sample from D0. The 16S rRNA amplicons of single clones were sequenced, and we found that these clones belonged to *Escherichia-Shigella* (data not shown). As expected, the overall diversity and richness of the bacterial microbiome were depleted both stably and continuously (Fig. 3 b, c). The Shannon diversity index showed that the diversity of the bacterial microbiome was significantly decreased after treatment with antibiotics and remained at a low level (Fig. 3B, Additional file 2: Figure S2C). The richness of the bacterial microbiome was significantly decreased, as measured by the ACE (Fig. 3c, Additional file 2: Figure S2D). In addition, there were noticeable differences in bacterial β-diversity between control and experimental animals, as determined using principal component analysis (PCA), and the results showed good repeatability within a single group (Additional file 2: Figure S2F).

Together, these data suggest that the richness and diversity of the bacterial microbiome composition were depleted stably and continuously. Therefore, we assessed the virome composition in the control and antibiotic-treated experimental monkeys on the ninth day.

### The virome community composition changed noticeably after treatment with antibiotics

In previous experiments, examination of microbiota genomes from rhesus macaques (*Macaca mulatta*) showed that a majority of the sequences in the fecal samples were mapped to bacterial genomes, while the percentage of sequences mapped to viral genomes was very low [20]. To comprehensively detect both DNA and RNA viruses, we filtered the fecal samples with a 0.45-μm filter and treated the samples with DNase I, after which, total DNA and RNA were extracted separately from the same fecal sample. Owing to low yield, amplification of the DNA and double-stranded cDNA by whole-genome amplification (using MDA) was necessary. Using the MiSeq 2 × 250 paired-end protocol on the Illumina MiSeq platform, we obtained an average of 54,590,439 ± 14,898, 536 clean reads per MDA sample library and generated a total of 88,610 Mbp from 12 samples, allowing detailed investigation of the viral populations. To catalog the present genes, we predicted open reading frames (ORFs). A total of 478,694 ORFs were predicted; the average ORF length was 398.1603279 bp, with a maximum of 16,635 bp and a minimum of 100 bp. The ecological signatures of the intestinal virome have been characterized. The largest percentage of sequences mapped to viromes in the fecal samples belonged to bacteriophages, accounting for over 80% of the sequences (Additional file 5: Table S2). We also identified eukaryotic DNA viruses and RNA viruses, as well as other viral families that are defined as “unclassified” in the NCBI Taxonomy database. In the raw data from the virome metagenomic analysis, RNA virus sequences were found in the DNA virus results, and DNA virus sequences were found in the RNA virus results, because the RNA virus sequences were wrongly affiliated with DNA viral genomes and vice versa. In addition, DNA viruses, especially bacteriophages, were the main components of the virome in our sequencing data, and the RNA from these viruses may have been extracted together with RNA viruses and vice versa. Thus, we excluded these data.

**Fig. 4:**
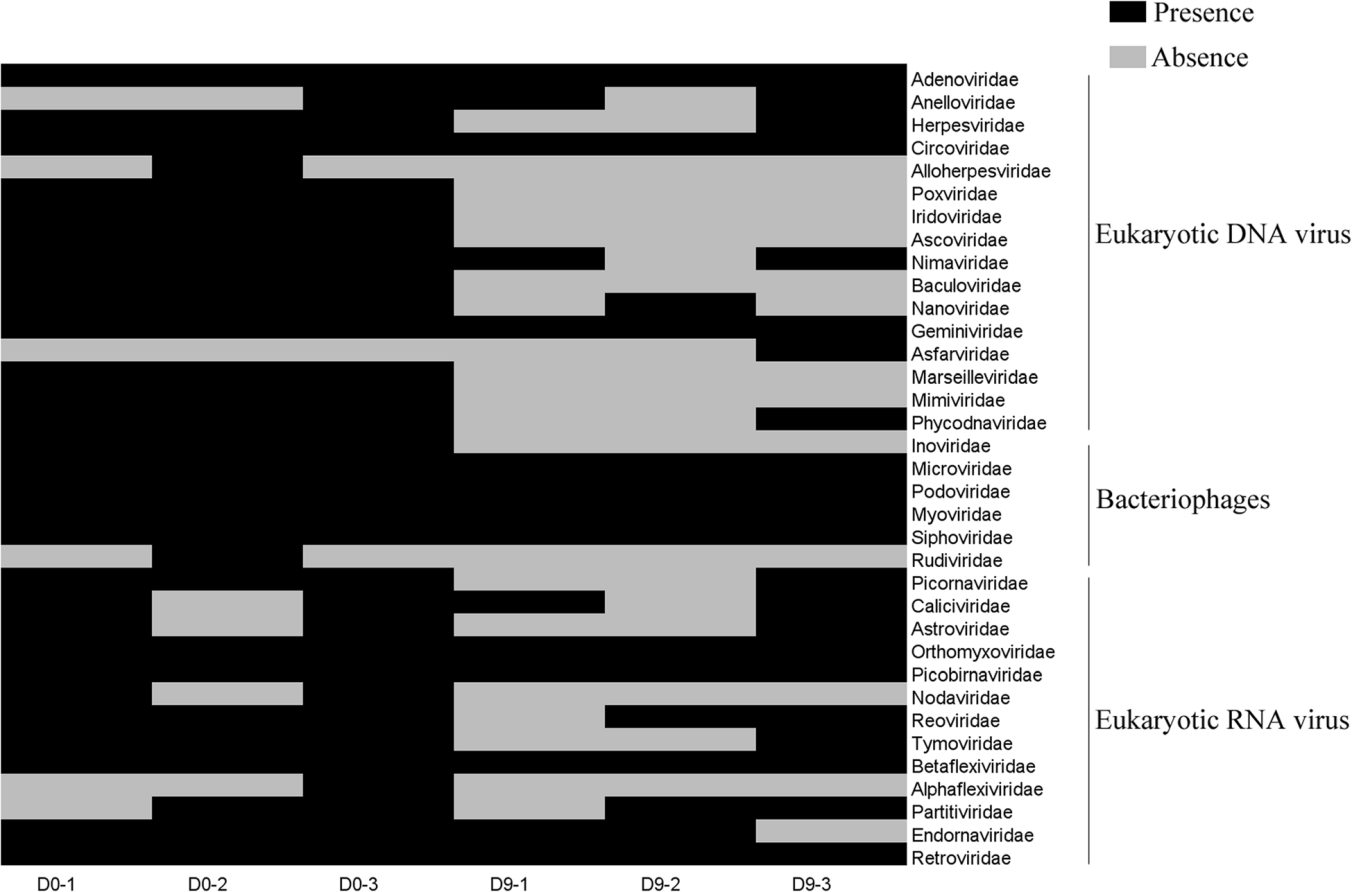
The composition of the virome changed noticeably after treatment with antibiotics. The presence-absence heatmap shows the virome characterized by metagenomic analysis. Due to the presence of low-complexity/repetitive regions in the reads, false-positive virus family taxonomic assignments with fewer than 3 reads were omitted from the analyses [26]

We observed that the fecal virome composition was noticeably altered after depletion of the bacterial microbiome treated with antibiotics. ANOSIM showed that the distance between groups was greater than that within groups for DNA viruses and bacteriophages (Additional file 6: Figure S4). DNA viruses, including members of the families *Poxviridae*, *Iridoviridae*, *Ascoviridae*, *Baculoviridae*, *Marseilleviridae*, and *Mimiviridae* and bacteriophages, such as members of the family *Inoviridae,* were present in the normal monkeys but absent after the gut bacterial microbiome was depleted (Fig. 4). DNA viruses, including members of the families *Herpesviridae*, *Nanoviridae*, and *Phycodnaviridae*, were present in three biological replicates before antibiotic treatment but in only one biological replicate after the gut bacterial microbiome was depleted (Fig. 4). Most of the reads from bacteriophages were noticeably depleted after the gut bacterial microbiome was depleted (Additional file 7: Figure S5, Additional file 8: Table S3). RNA viruses, including members of the families *Picornaviridae* and *Tymoviridae*, were present in three biological replicates but were present in only one biological replicate after the gut bacterial microbiome was depleted, and RNA viruses belonging to *Nodaviridae* were present in two biological replicates but absent after the gut bacterial microbiome was depleted (Fig. 4). In addition, many kinds of viral groups, including *Circoviridae*, *Geminiviridae*, *Microviridae*, *Podoviridae*, *Myoviridae*, *Siphoviridae*, *Picornaviridae*, and *Retroviridae*, were present regardless of whether the bacterial microbiota was depleted, but the sequencing reads showed that the abundances of these viruses may have decreased with bacterial microbiota depletion (Additional file 7: Figure S5). However, we could not validate this decrease because the data from deep sequencing were only qualitative, not quantitative.

### Depletion of the virome at the species level was validated by real-time PCR

**Fig. 5:**
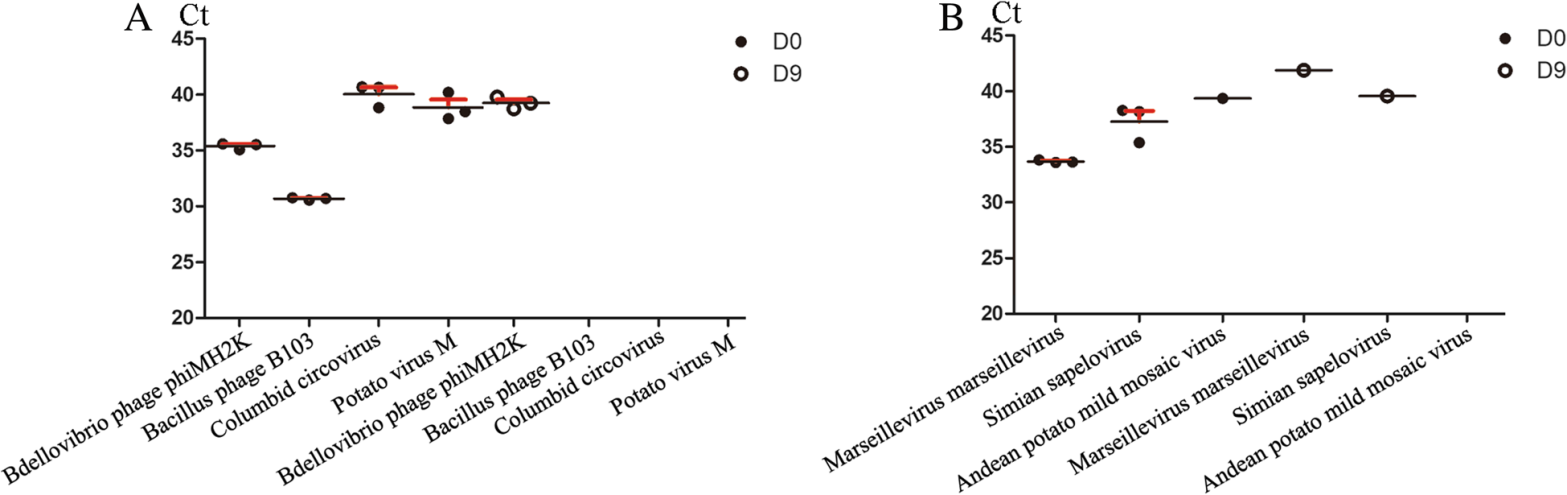
Depletion of viromes at the species level after depletion of the gut bacterial composition validated by real-time PCR. (**a**) As the templates, the DNA and cDNA extracted from fecal samples were detected directly by real-time PCR. (**b**) The samples that were not detected directly from DNA or cDNA were subjected to MDA and then used as templates for detection by real-time PCR. We used the Ct numbers to show viral richness. The black line represents the mean of 3 replicates, and the red line represents the SEM. The samples that were not detected are not shown

Regarding the abundances of viruses at the species level, the results that were similar in 2 or more monkeys were selected, and we used real-time PCR to validate changes in the richness of the viruses. As the figure shows, we analyzed fecal samples that had not been subjected to MDA. After the bacterial microbiome was depleted, the results from real-time PCR validated the depletion of 4 viral species: *Bdellovibrio phage phiMH2K*, which belongs to the *Microviridae* family of bacteriophages; *Bacillus phage B103*, which belongs to the *Podoviridae* family of bacteriophages; *columbid circovirus*, which belongs to the *Circoviridae* family of DNA viruses; and *potato virus M*, which belongs to the *Betaflexiviridae* family of RNA viruses (Fig. 5a). The samples that were not detected directly from DNA or cDNA were subjected to MDA and then detected by real-time PCR. The depletion of 3 viral species was detected (Fig. 5b): *Marseillevirus marseillevirus*, which belongs to the *Marseilleviridae* family of DNA viruses; *simian sapelovirus*, which belongs to the *Picornaviridae* family of RNA viruses; and *Andean potato mild mosaic virus*, which belongs to the *Tymoviridae* family of RNA viruses. In addition, *Mason-Pfizer monkey virus*, which belongs to the *Retroviridae* family, was detected among the RNA viruses; this virus is very dangerous in monkey populations and has been shown to cause an AIDS-like disease in rhesus macaques [33]. Encouragingly, no *Mason-Pfizer monkey virus* was detected by real-time PCR.

Briefly, the fecal virome composition was noticeably altered after depletion of the bacterial microbiome, and the abundances of many DNA viruses, bacteriophages and RNA viruses in the gut were clearly decreased. In addition, in the metagenomic analysis, we found high numbers of reads from DNA viruses and bacteriophages; however, low numbers of reads from RNA viruses were found (Additional file 7: Figure S5, Additional file 8: Table S3). These results may be due to the limited application of MDA in RNA viruses.

### Shifts in the virome were correlated with deletion of the bacterial microbiome

**Fig. 6:**
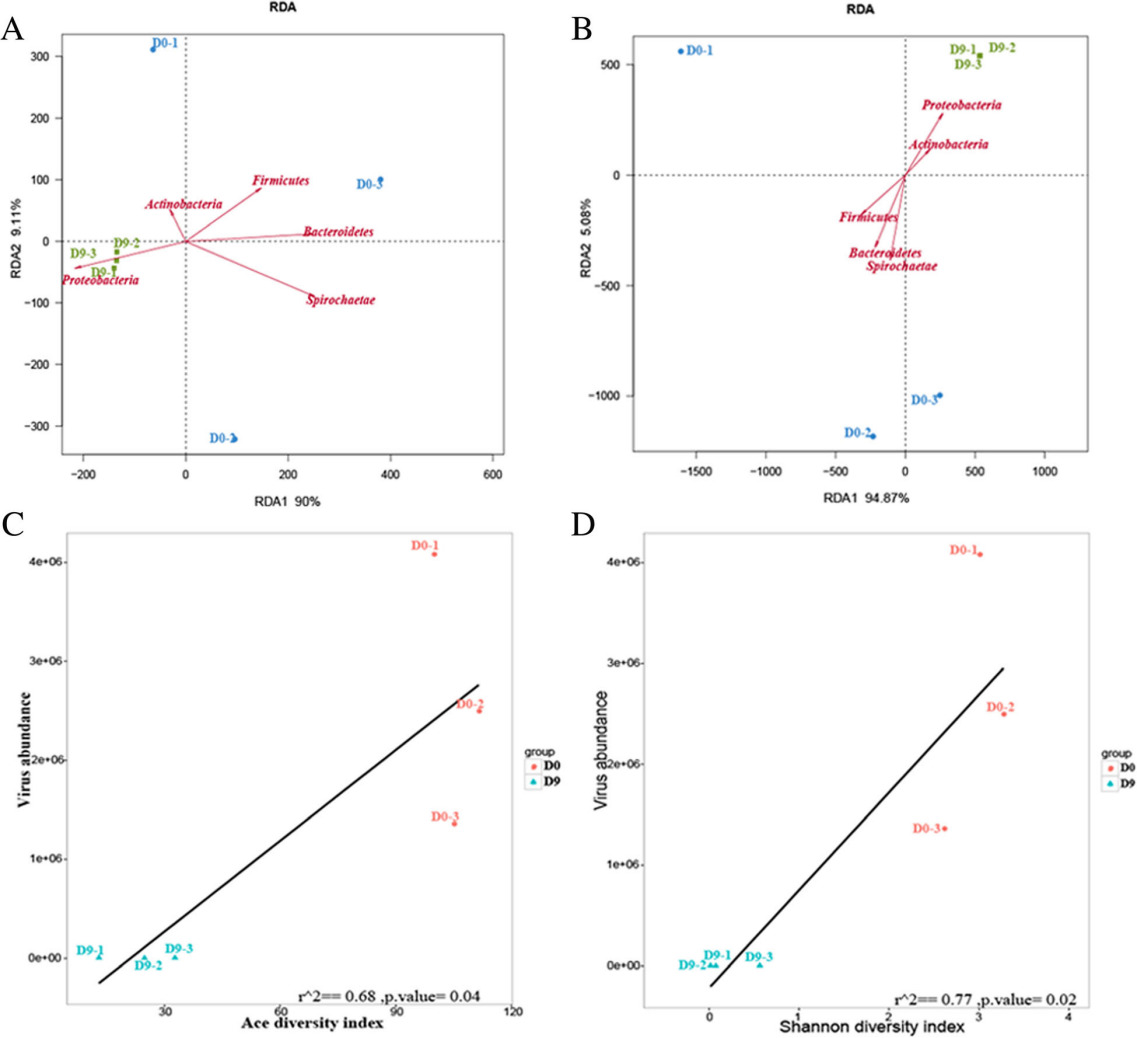
The abundance of the virome was correlated with the bacterial composition. (**a**, **b**) RDA of DNA viruses and bacteriophages, using abundances of the bacterial microbiome at the phylum level as environmental factors. Red arrows represent the digital environmental factors. If the sample point is in the direction of the arrow, there is a positive interaction between the environmental factor and the sample distribution, and if the sample point is in the opposite direction as the arrow, there is a negative interaction between the environmental factor and the sample distribution. The length of each arrows represents the degree of impact. (**c**, **d**) Linear regression analysis between bacteriophage abundance and both ACE and Shannon diversity index. We found positive correlations in both cases (*p* < 0.05)

The microbiota structure is the result of dynamic interactions among various community members. We found a close interaction between the whole virome (DNA viruses, RNA viruses and bacteriophages) and the bacterial microbiome. We analyzed the effects on the abundance of the virome by RDA, taking the richness of bacteria at the phylum level as environmental factors, and found a negative interaction between the abundances of DNA viruses and bacteriophages at D9 and the abundances of most bacteria (Fig. 6a, b). However, RNA viruses exhibited chaotic interactions due to weak repeatability. Next, we conducted linear regression analysis between virome abundance and both the ACE and Shannon diversity index, and we found positive correlations in bacteriophages (Fig. 6c, d). In addition, the DNA and RNA viruses showed a positive trend (Additional file 9: Figure S6) but weak confidence levels.

Overall, these results support our hypothesis that a clear interomic relationship exists between the virome and bacterial microbiome. Positive correlations were found between virome abundance and the richness and diversity of the bacterial microbiome.

### Metabolites produced by the bacterial microbiome shifted noticeably and could inhibit or promote the survival of viruses

**Fig. 7:**
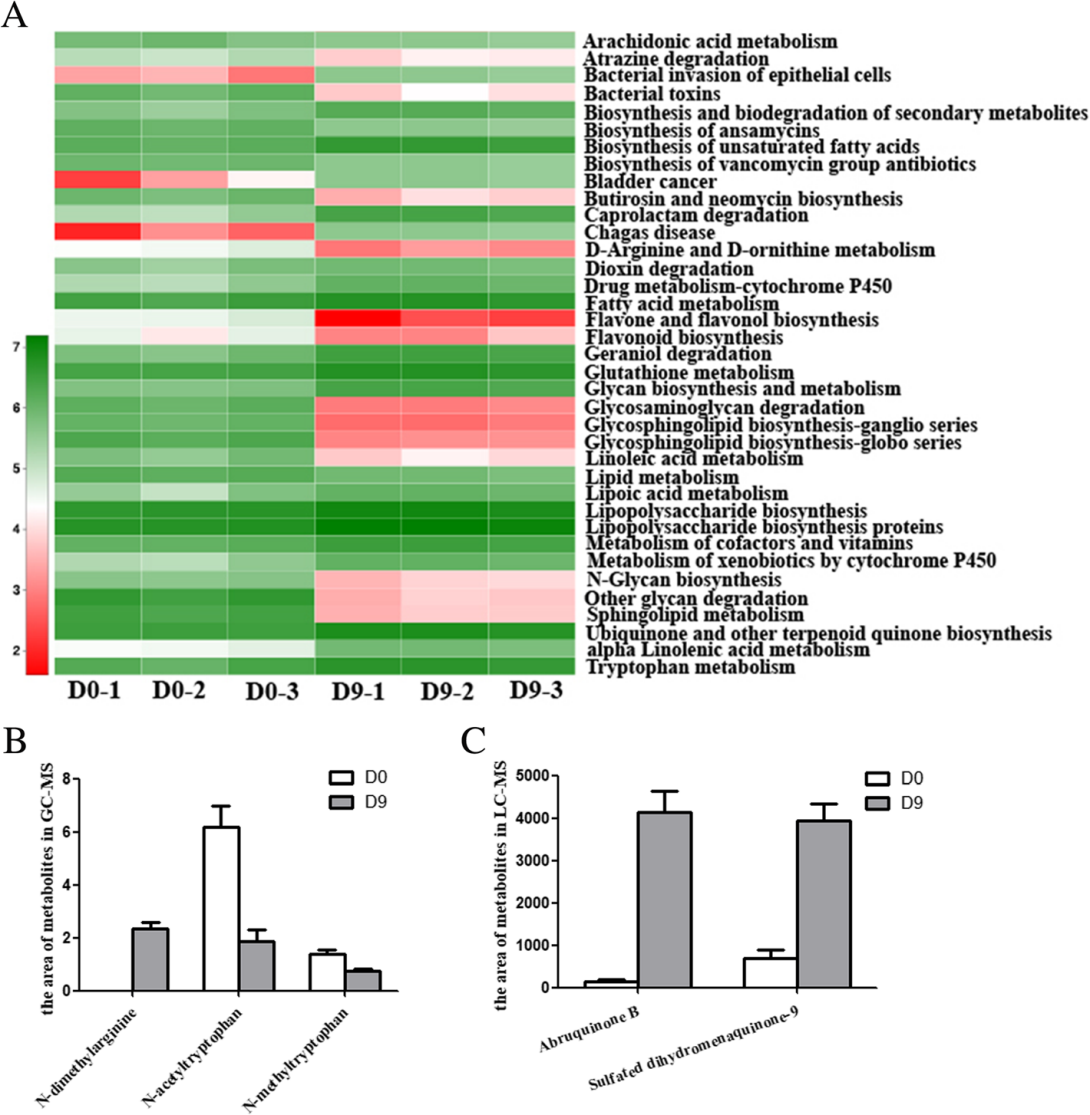
Metabolites produced by the bacterial microbiome shifted noticeably and could inhibit or promote the survival of viruses. (**a**) Heatmap of the richness of metabolites in KEGG predicted by normalization of OTUs in the bacterial microbiome using PICRUSt. The numbers in the heatmap are the log values of metabolite richness. (**b**, **c**) The metabolites detected by LC-MS and GC-MS were consistent with the predictions from the KEGG analysis. The number represents the integral value of the peak area

Metabolites could inhibit or promote viruses in vivo [34] and in vitro [35–37], and the metabolites produced by bacteria play important roles in host physiology [38]. To interrogate the functions associated with the response to depletion in the bacterial microbiome, we performed a KEGG prediction analysis of the bacterial microbiome using PICRUSt. We observed significant differences in functional systems along with shifts in the composition of the bacterial microbiome, according to the predictions by PICRUSt. KEGG pathways associated with bacterial toxins were downregulated significantly (Fig. 7a), perhaps as a result of antibiotic treatment. The pathway associated with D-arginine metabolism showed a 9-fold decrease; in contrast, the pathways associated with fatty acid metabolism and tryptophan metabolism showed a 2-fold and 2.6-fold increase, respectively. At the same time, the biosynthetic pathway for ubiquinone and other terpenoidquinones showed a 2-fold increase. Moreover, the glycosaminoglycan degradation pathway exhibited low diversity and a 1300-fold decrease (Fig. 7a). Glycan [39], glycosaminoglycan [40], quinone [41] and arginine [42, 43] are well known to support the inhibition of viruses, while tryptophan [44, 45] and fatty acids [36] promote viral survival.

Interestingly, we detected the metabolites in the fecal samples by metabolome scanning and found that the changes in some metabolite levels were consistent with our prediction based on the normalization of OTUs in the 16S rRNA amplicon sequencing data. Because metabolomic analysis requires 6 biological duplications, the metabolomes of 6 samples collected before treatment with antibiotics and those of samples collected after treatment with antibiotics for 8 and 9 days were examined by GC-MS and LC-MS. The metabolomic results exhibited good repeatability (Additional file 10: Figure S7). In GC-MS (Fig. 7b), N-dimethylarginine was not detected in monkeys that were not treated with antibiotics, but the metabolite was present after antibiotic treatment. The levels of N-acetyltryptophan and N-methyltryptophan showed a 3.3-fold and 1.85-fold decrease, respectively, after treatment with antibiotics. In LC-MS (Fig. 7c), we found that the levels of abruquinone B and sulfated dihydromenaquinone-9 exhibited a 30-fold and 5.6-fold increase, respectively. Regrettably, in the present study, we did not measure the levels of glycosaminoglycan, which plays a very important role in reducing the viral population [40].

## Discussion

As reported by Adina Howe, Yatsunenko T and Alejandro Reyes, in the same environment and feeding conditions, the composition of the microbiota and virome could remain stable within an individual [17, 46, 47]. However, the gut microbial composition could be influenced by multiple interacting factors, such as diet [46], antibiotic use [48], age, geographical setting [47], and several diseases, including chronic inflammation, obesity and diabetes [4]. In our study, the major reason for depletion of the gut bacterial microbiota was treatment with the antibiotic cocktail. The feeding conditions of the rhesus monkeys were stable in terms of their food and water consumption, and blood samples were monitored routinely, showing that there was no infection during the study period (Additional file 1: Figure S1). The bacterial composition exhibited stable and continuous depletion after treatment with the antibiotic cocktail, and we found that the virome composition changed noticeably and was correlated with the shifts in the bacterial community. Moreover, we found that metabolites produced by the gut bacterial microbiome may play a role in the interrelation. In addition, we found that the composition of the rhesus monkey enterovirus group was similar to that of the human enterovirus group [26], and our results may be beneficial for research on the composition of the human virome.

When the bacterial microbiome was depleted, ampicillin could kill most bacteria, including gram-positive and gram-negative bacteria; streptomycin could kill most bacilli; kanamycin could kill most gram-negative bacteria; metronidazole could kill most anaerobic bacteria and parasites; and vancomycin could kill most gram-positive bacteria. Of course, the numbers of drug-resistant bacteria are increasing, but we believe that the cocktail of five antibiotics could deplete most of the commensal bacteria in the gut. As expected, the whole gut bacterial microbiome, including gram-positive and gram-negative bacteria (Additional file 2: Figure S2E), was depleted after treatment with the antibiotic cocktail, except for *Escherichia*-*Shigella* species belonging to Proteobacteria, which were resistant to the cocktail. *Escherichia* harbored the most diverse antibiotic resistance genes, including genes resistant to multidrug treatments, tetracycline, aminoglycoside, macrolide-lincosamide-streptogramin B, β-lactams, and sulfonamides [49]. We maintained the bacteria belonging to *Escherichia-Shigella* in plates with the antibiotic cocktail. In the future, we will investigate the specific resistance and antibiotic resistance genes in this bacterium. Notably, our study focused on the interaction between virome composition and the bacterial microbiome in rhesus monkeys and may serve as a model for gut microbiota analysis. Therefore, we used the administration of 5 distinct antibiotics at high dosages and high frequencies for 2 weeks to deplete the whole gut bacterial microbiome. In our results, the richness and diversity of the bacterial community were depleted. Because our study did not involve clinical treatment, the normal dose of antibiotics was not evaluated by our procedure.

People are widely prescribed antibiotics each year [50], and while antibiotics exert very complex effects on the whole bacterial microbiome [48], the effects of these drugs on the virome are not clear. Antibiotics can directly affect viruses but do not exhibit a wide range of roles. Minocycline [51], berberine, abamectin, ivermectin [52], glycopeptides [53], and teicoplanin [54] could inhibit the corresponding viruses. In our study design, an antibiotic cocktail that included ampicillin, streptomycin, kanamycin, metronidazole, and vancomycin was administered orally. No study has yet reported that these antibiotics directly affect viruses.

Based on our results, the richness of these viruses was very low in the gut, and we had to use MDA to perform deep sequencing, although the detection by deep sequencing was very sensitive. We first characterized the shift in the gut virome by deep sequencing, and the samples were amplified by MDA. MDA is used as a general technique in virome research, especially for DNA virome detection [26]. To a certain extent, the amplification read-out can also represent the virus quantity. However, MDA is not well suited to the detection of RNA viruses. The sequence-independent amplification (SIA) approach is more appropriate than MDA for detecting RNA viruses [55]. In the future, we can use this approach to precisely detect RNA viruses. In this case, we validated the depletion of the virome composition, including DNA viruses, RNA viruses and bacteriophages, by real-time PCR. Although the number of cycles seemed high, these results were verified via three biological replicates, and the results of the no-template control (NTC) were not detected. In addition, we performed serial dilution of the in vitro transcribed RNA of coxsackievirus A16 to generate a standard curve and found that a Ct of 39.96 represents 23 genomic copies (data not shown). In our opinion, these viruses are components of the gut microbiome with low richness and may be involved in host physiology.

The metabolites produced by gut bacteria play very important roles in host physiology [38], although the effects of these metabolites on virome composition have rarely been reported. Glycan [39], glycosaminoglycan [40], quinone [41] and arginine [42, 43] support the inhibition of viruses, while tryptophan [44, 45] and fatty acids [36] promote viral survival. Although most metabolites that can inhibit or promote viruses play roles in human viruses, tryptophan could promote the simian immunodeficiency virus in macaques [45]. In addition, most pandemics originating in animals, such as severe acute respiratory syndrome and pandemic influenza, could start to appear because of ecological, behavioral, or socioeconomic changes [56]. Many human viruses are zoonotic, and some human viruses, such as human enterovirus 71, can infect animals, especially monkeys [32]. We believe that metabolites play roles in a broad spectrum of viruses and that changes in the metabolites may correlate with depletion of the virome. In our results, the level of quinone, which decreases the abundance of viruses, was increased in the gut metabolome, and the levels of some amino acids that promote the survival of viruses, such as tryptophan, were decreased. Importantly, glycosaminoglycan, which can reduce the populations of various viruses, was noticeably increased in the KEGG pathways of the bacterial microbiome, but we did not measure glycosaminoglycan levels in the present study. It is very difficult to detect glycosaminoglycan by metabolic scanning because glycosaminoglycan has a very high molecular weight. In the future, glycosaminoglycan levels could be measured by time-of-flight mass spectrometry. First, the polysaccharide needs to be dispelled, followed by detection of the monosaccharide to calculate the polysaccharide levels based on the relationships among the monosaccharides in a specific database. However, this process is very complicated, and the database is not sufficiently large at present. By analyzing the relevant data, we found that depletion of bacteria directly promoted changes in the concentrations of some metabolites, which may play important roles in reducing the abundance of DNA viruses.

## Conclusion

Our metagenomic-scale characterization of the virome composition after treatment with antibiotics supports the notion that the composition of the virome is noticeably altered in correlation with bacterial community depletion and that metabolites produced by bacteria possibly play important roles in the interaction. The next step will be to investigate the underlying mechanisms in detail.

## Additional files

Additional file 1: **Figure S1.** The detection of blood cell analysis during the course of antibiotic treatment. White blood cells (WBC), neutrophilic granulocytes (NEUT), lymphocytes (LYMPH), monocytes (MONO#), eosinophils (EO#), and basophilic granulocytes (BASO#) were counted, and the counts were compared between the monkeys that were treated with antibiotics and ones were not, and there was no obvious difference. (TIF 597 kb)

Additional file 2: **Figure S2.** The richness and diversity of gut bacterial microbiota were depleted obviously stably and continuously. (A, B) The community analysis of gut bacterial microbiota on phylum level. The phylum was represented by own color.(C, D) The student’s t-test of Alpha diversity index (the Shannon diversity index and the ACE estimator) in genus level. 0.01 < *P* ≤ 0.05 was marked *, 0.001 < *P* ≤ 0.01 was marked * *, *P* ≤ 0.001 was marked * * * .(E)The longitudinally reads of OTU in gram-positive and gram-negative bacteria. (F) The repeatability analysis of 16S rRNA amplicon sequencing by PCA. (TIF 978 kb)

Additional file 3: **Table S1.** The reads number on the genus level in the gut bacterial communities in a longitudinal cohort treated with an antibiotic cocktail. (XLS 22 kb)

Additional file 4: **Figure S3.** The phylogenetic tree on genus level of gut bacterial microbiome. The number in the line represents the genetic distance. Every phlym was showed in own color. The bar in the right were caculated according to the number of reads. (TIF 966 kb)

Additional file 5: **Table S2.** The reads number on the family level in the gut virome communities in a longitudinal cohort treated with an antibiotic cocktail. (XLS 2 kb)

Additional file 6: **Figure S4.** The Anosim analysis of virome groups. The ordinate represents the distance value. R value represents the statistic results, and the closer the R value is to 1, the greater the difference between groups than the difference in the group, and the grouping was reasonable. (TIF 495 kb)

Additional file 7: **Figure S5** (A) Heatmap of abundance of the DNA virus composition on the family level. (B) Heatmap of abundance of the bacteriophages composition on the family level. (C) Heatmap of abundance of the RNA virus composition on the family level. The hosts that belong to the same domain are shown in the same color on the left. The abundance were represented by the summation of the reads number and the contigs numbers which removed the repeated number with corresponding reads, and the color bar showed the summation gradient. The results which were similar in 2 and more than 2 monkeys were analyzed in our results. (TIF 723 kb)

Additional file 8: **Table S3.** The reads number on the family level belong to bacteriophages and the eukaryotic viruses including DNA viruses and RNA viruses. (XLS 27 kb)

Additional file 9: **Figure S6.** The linear regression analysis between virome abundance and Ace estimator index, the Shannon diversity index. (A, B) The linear regression analysis between DNA virome abundance and Ace estimator index, Shannon diversity index. (C, D) The linear regression analysis between RNA virome abundance and Ace estimator index, Shannon diversity index. (TIF 441 kb)

Additional file 10: **Figure S7.** The Principal Component Analysis analysis of metabolome detecting by PCA. (A) PCA analysis of GC-MS. (B) PCA analysis of LC-MS. (TIF 261 kb)

## Data Availability

All data generated or analyzed during this study are included in this published article and the additional files. We have deposited the raw sequencing data into NCBI and the number is PRJNA555120.

## Abbreviations

ACE: Abundance-based coverage estimator
bp: Base pair
CFU: Colony-forming unit
COG: Cluster of Orthologous Groups of proteins
Ct: Cycle threshold
GC-MS: Gas chromatography-mass spectrometry
KEGG: Kyoto Encyclopedia of Genes and Genomes
KO: KEGG Ortholog
LC-MS: Liquid chromatography-mass spectrometry
MDA: Multiple displacement amplification
NTC: No-template control
ORF: Open reading frame
OTU: Operational taxonomic unit
PCA: Principal component analysis
PICRUSt: Phylogenetic investigation of communities by reconstruction of unobserved states
RDA: Redundancy analysis
SIA: Sequence-independent amplification
