# Supplementary figures and images for: The altered gut virome community in rhesus monkeys is correlated with the gut bacterial microbiome and associated metabolites
Source: Virol J. 2019 Aug 19;16:105. doi: 10.1186/s12985-019-1211-z (PMC6700990; doi:10.1186/s12985-019-1211-z)

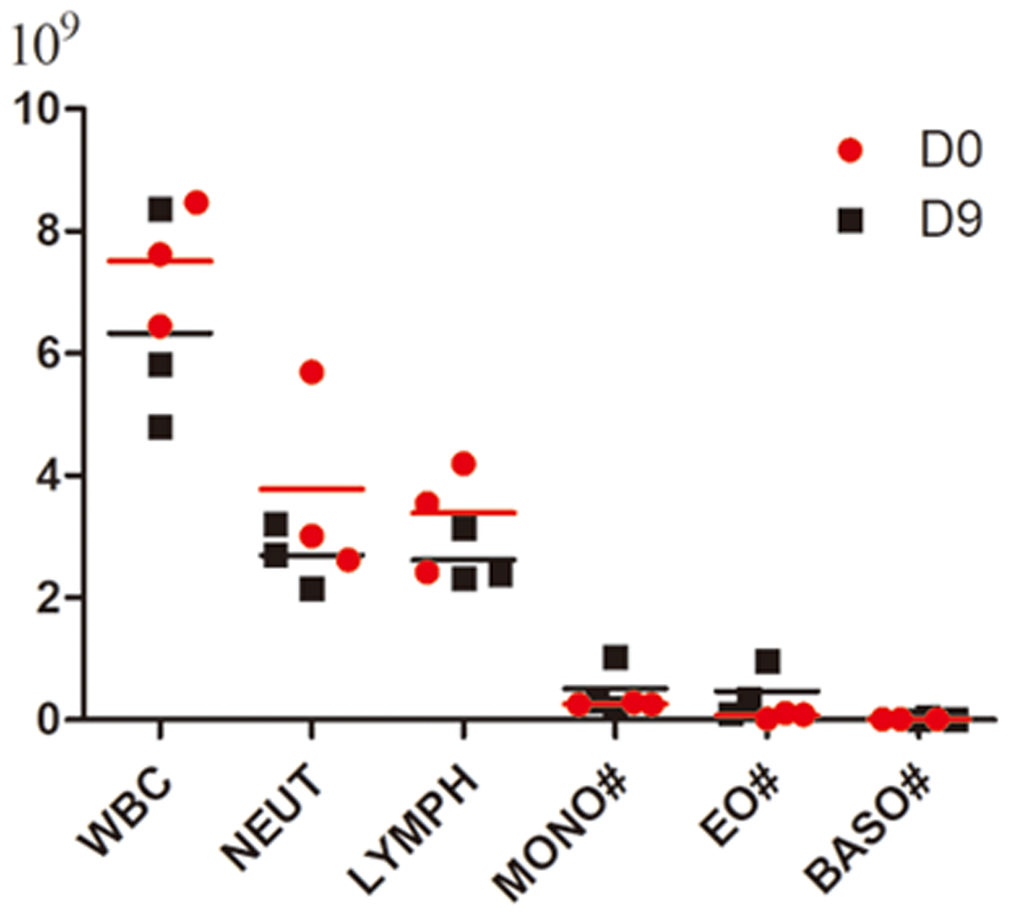

Supplement: Supplementary file 1 — Figure S1. The detection of blood cell analysis during the course of antibiotic treatment. White blood cells (WBC), neutrophilic granulocytes (NEUT), lymphocytes (LYMPH), monocytes (MONO#), eosinophils (EO#), and basophilic granulocytes (BASO#) were counted, and the counts were compared between the monkeys that were treated with antibiotics and ones were not, and there was no obvious difference. (TIF 597 kb) [file 12985_2019_1211_MOESM1_ESM.tif]

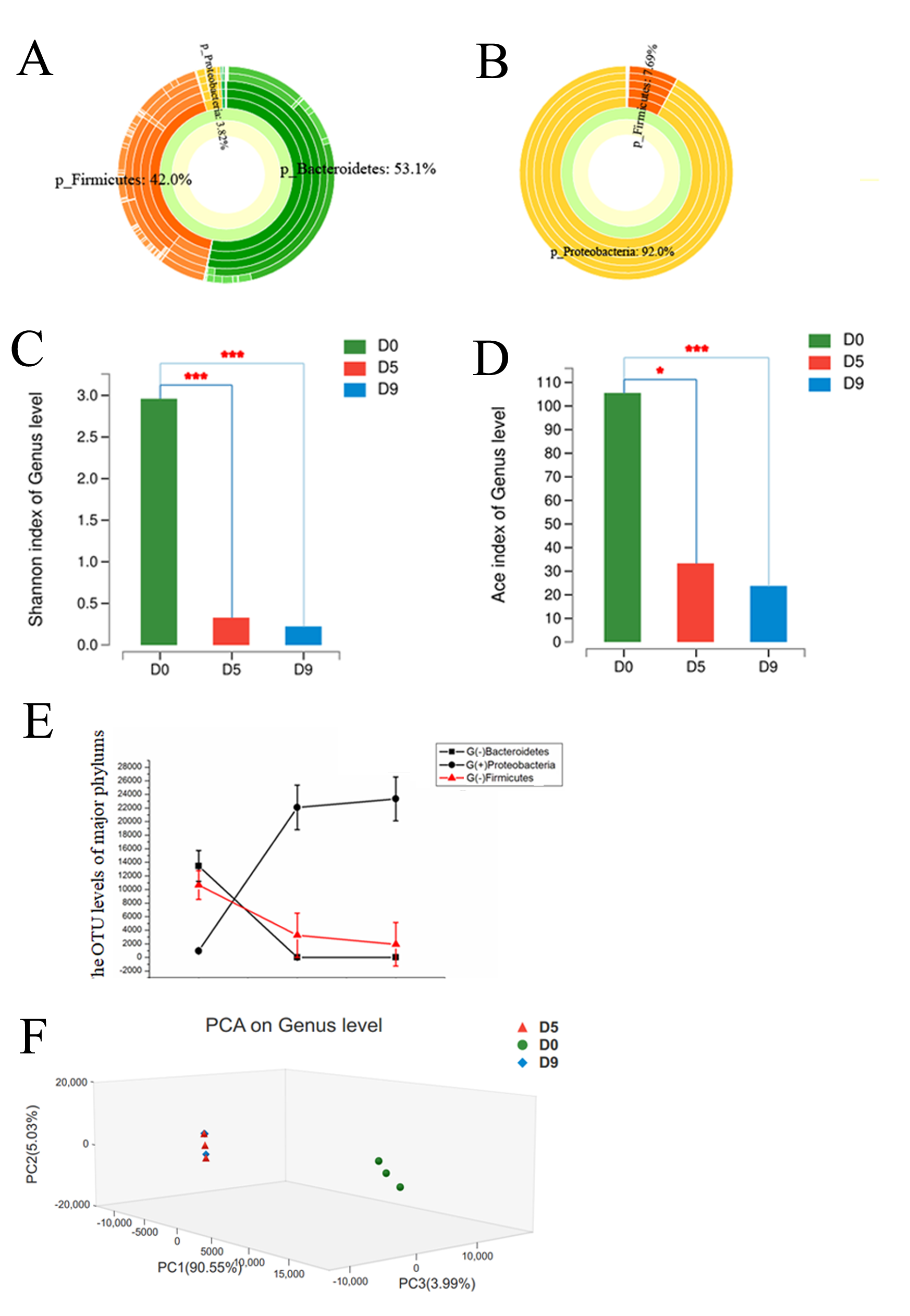

Supplement: Supplementary file 2 — Figure S2. The richness and diversity of gut bacterial microbiota were depleted obviously stably and continuously. (A, B) The community analysis of gut bacterial microbiota on phylum level. The phylum was represented by own color.(C, D) The student’s t-test of Alpha diversity index (the Shannon diversity index and the ACE estimator) in genus level. 0.01 < P ≤ 0.05 was marked *, 0.001 < P ≤ 0.01 was marked * *, P ≤ 0.001 was marked * * * .(E)The longitudinally reads of OTU in gram-positive and gram-negative bacteria. (F) The repeatability analysis of 16S rRNA amplicon sequencing by PCA. (TIF 978 kb) [file 12985_2019_1211_MOESM2_ESM.tif]

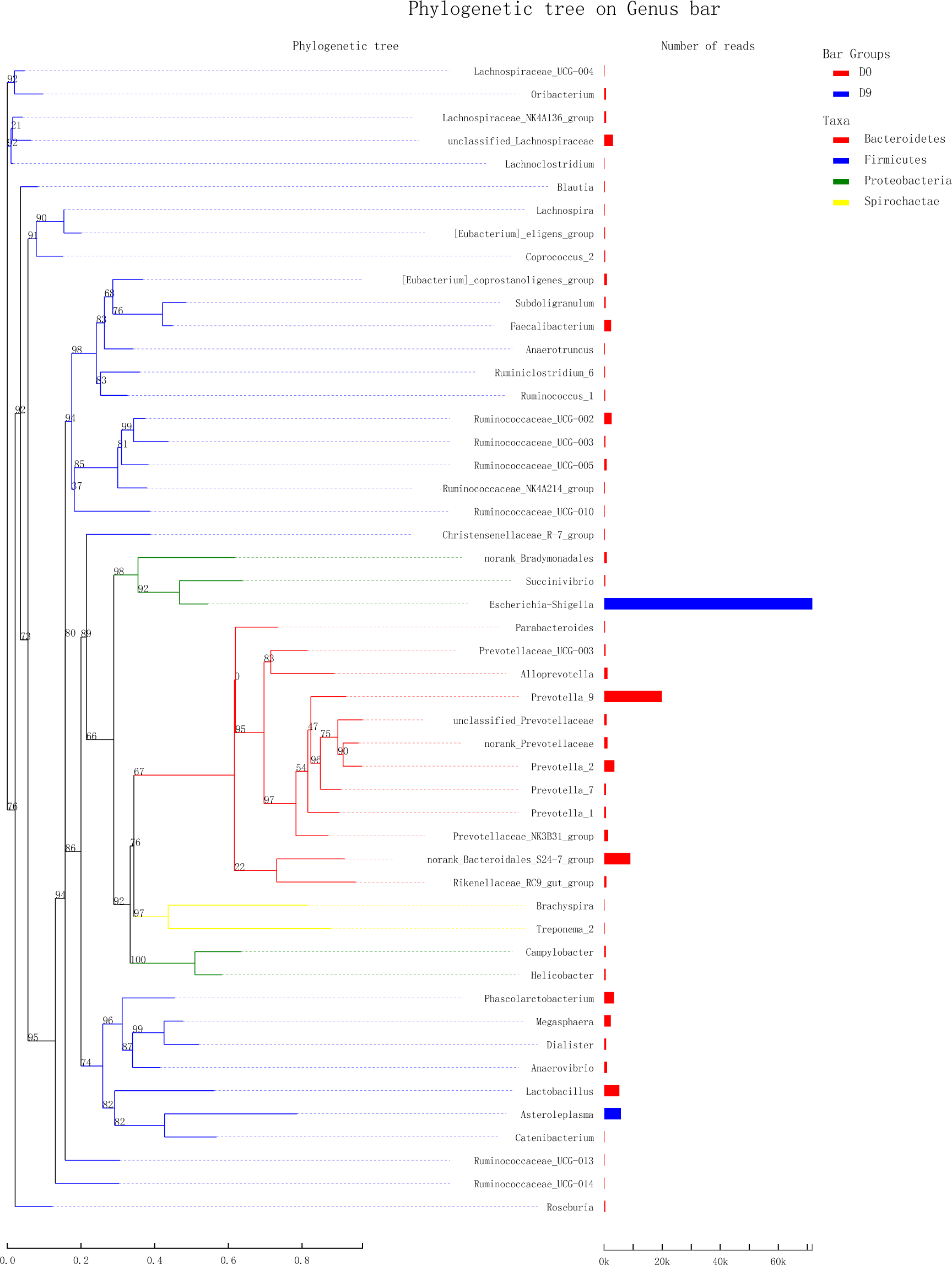

Supplement: Supplementary file 4 — Figure S3. The phylogenetic tree on genus level of gut bacterial microbiome. The number in the line represents the genetic distance. Every phlym was showed in own color. The bar in the right were caculated according to the number of reads. (TIF 966 kb) [file 12985_2019_1211_MOESM4_ESM.tif]

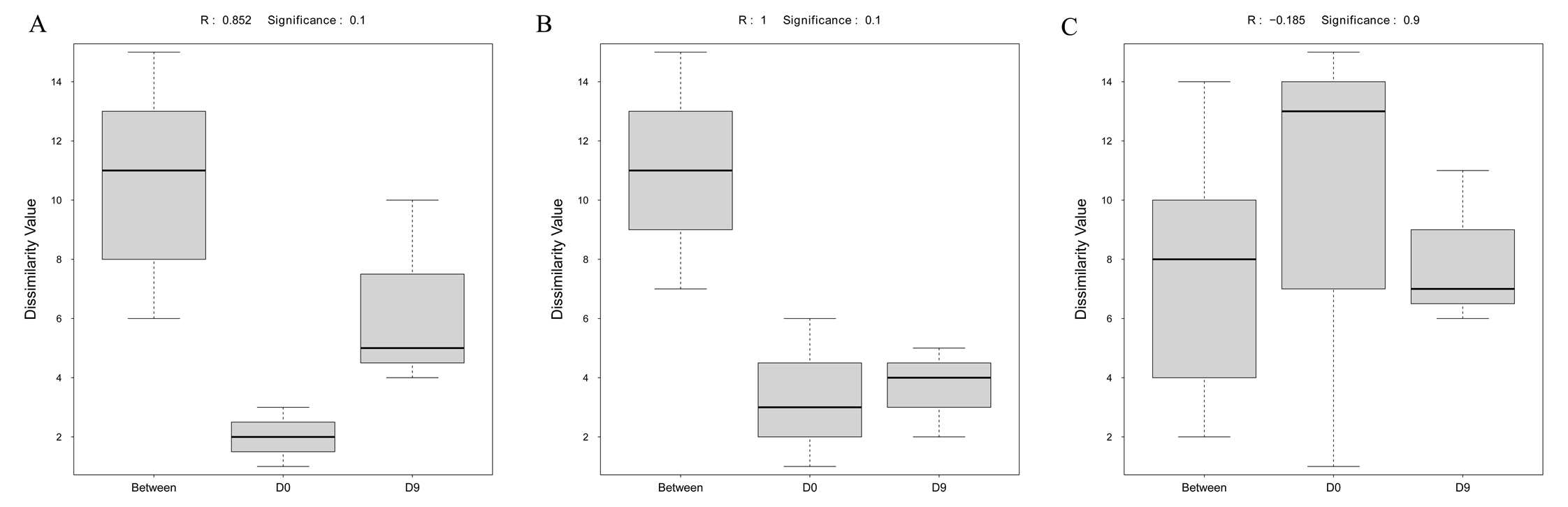

Supplement: Supplementary file 6 — Figure S4. The Anosim analysis of virome groups. The ordinate represents the distance value. R value represents the statistic results, and the closer the R value is to 1, the greater the difference between groups than the difference in the group, and the grouping was reasonable. (TIF 495 kb) [file 12985_2019_1211_MOESM6_ESM.tif]

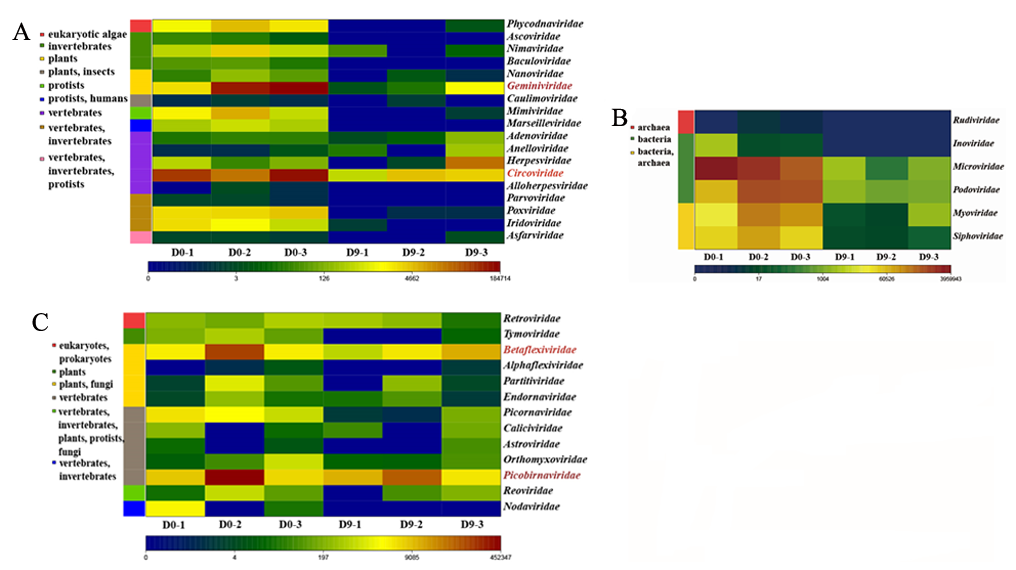

Supplement: Supplementary file 7 — Figure S5 (A) Heatmap of abundance of the DNA virus composition on the family level. (B) Heatmap of abundance of the bacteriophages composition on the family level. (C) Heatmap of abundance of the RNA virus composition on the family level. The hosts that belong to the same domain are shown in the same color on the left. The abundance were represented by the summation of the reads number and the contigs numbers which removed the repeated number with corresponding reads, and the color bar showed the summation gradient. The results which were similar in 2 and more than 2 monkeys were analyzed in our results. (TIF 723 kb) [file 12985_2019_1211_MOESM7_ESM.tif]

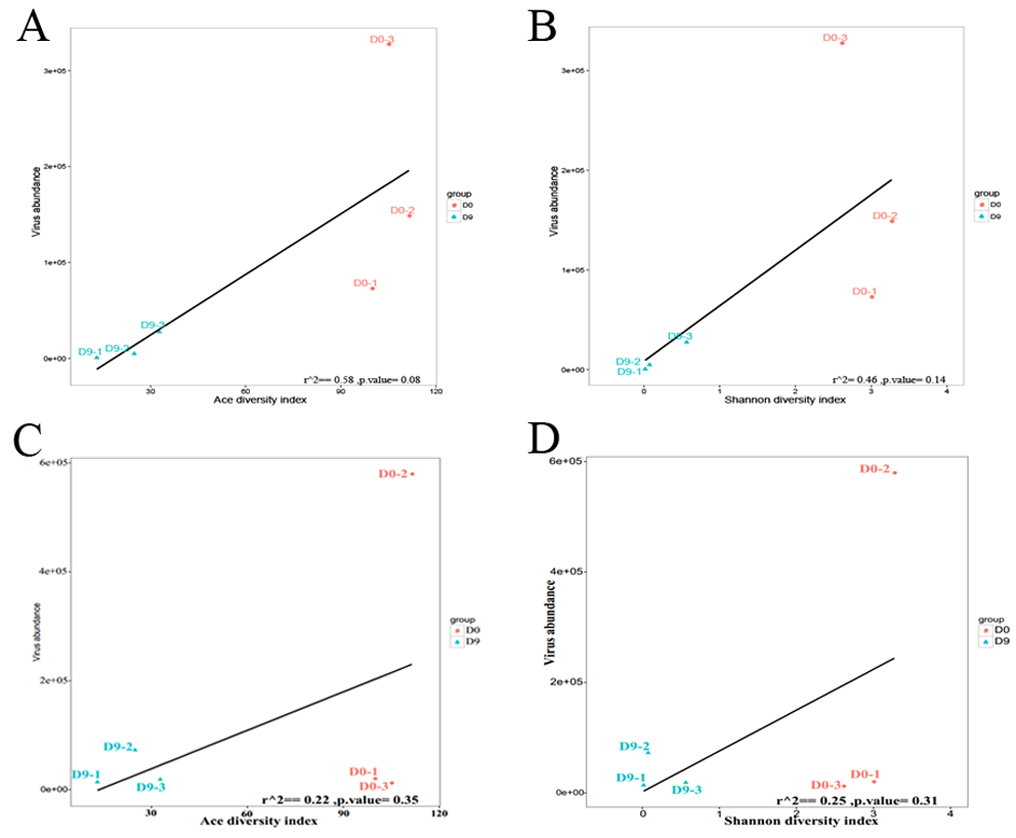

Supplement: Supplementary file 9 — Figure S6. The linear regression analysis between virome abundance and Ace estimator index, the Shannon diversity index. (A, B) The linear regression analysis between DNA virome abundance and Ace estimator index, Shannon diversity index. (C, D) The linear regression analysis between RNA virome abundance and Ace estimator index, Shannon diversity index. (TIF 441 kb) [file 12985_2019_1211_MOESM9_ESM.tif]

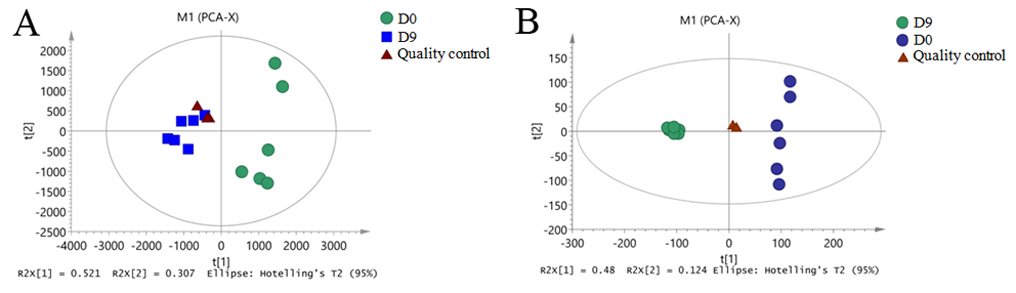

Supplement: Supplementary file 10 — Figure S7. The Principal Component Analysis analysis of metabolome detecting by PCA. (A) PCA analysis of GC-MS. (B) PCA analysis of LC-MS. (TIF 261 kb) [file 12985_2019_1211_MOESM10_ESM.tif]
